# Detection of Insertion/Deletions (InDel) Within Five Clock Genes and Their Associations with Growth Traits in Four Chinese Sheep Breeds

**DOI:** 10.3390/vetsci12010039

**Published:** 2025-01-09

**Authors:** Ziteng Wang, Xiaohua Yi, Mengzhe Yang, Xiuzhu Sun, Shuhui Wang

**Affiliations:** 1 College of Animal Science and Technology, Northwest A&F University, Yangling 712100, China; wangziteng@nwafu.edu.cn (Z.W.); yixiaohua@nwafu.edu.cn (X.Y.); yangmeng2018@nwafu.cn (M.Y.); 2 College of Grassland Agriculture, Northwest A&F University, Yangling 712100, China; sunxiuzhu208@163.com

**Keywords:** sheep, circadian clock gene, insertion/deletion (InDel), growth traits, association

## Abstract

Organisms have the capacity to detect day–night fluctuations through oscillators regulated by circadian clock genes, which are crucial for regulating various biological processes. Numerous studies have demonstrated a marked association between these genes and various growth traits of sheep. This study identified polymorphisms at 23 potential loci within five clock genes in four Chinese sheep breeds. Only two polymorphic insertion/deletions (InDels) were detected in *CLOCK* and *PER3* genes, respectively. The distribution of these two loci in four Chinese sheep breeds and their association with growth traits were further explored. A 12 bp deletion was found in the intron of the *CLOCK* gene (rs604230640), which was significantly associated with body height (*p* < 0.05), body oblique length (*p* < 0.05) and cannon girth (*p* < 0.05) in Hu sheep (HS). A 22 bp insertion in the intron of the *PER3* gene (rs600537720) with a dominant genotype of insertion/insertion (II) was found to have a significant association with chest depth (*p* < 0.05) in Small-Tail Han sheep (STHS), tail width (*p* < 0.05) in Tong Sheep (TS), and in Lanzhou fat-tailed sheep (LFTS). In conclusion, this study has elucidated the polymorphisms of *CLOCK* and *PER3* genes and has examined the influence of these two genes on the growth traits of sheep. Concurrently, the two molecular markers identified in *CLOCK* and *PER3* could potentially serve in the marker-assisted selection of growing-related traits in local Chinese sheep breeds.

## Simple Summary

Circadian clock genes are involved in and regulate many physiological processes in an organism. It is advantageous to find the potential molecular markers that are associated with the growth and development of sheep through molecular breeding. In this study, 23 loci of five clock genes were detected in four Chinese sheep breeds. We found two loci of the *CLOCK* gene and *PER3* genes, respectively, which were significantly associated with sheep growth traits. In conclusion, the two molecular markers in *CLOCK* and *PER3* could potentially be used for marker-assisted selection of growth traits in local Chinses sheep breeds. These novel findings may provide a theoretical basis for molecular breeding and genetic selection of sheep.

## 1. Introduction

Growth traits, which are key phenotypic traits reflecting growth, body structure, and the development of tissues and organs, are crucial indicators of the growth and development of meat sheep. These traits are closely associated with production performance, disease resistance and livestock adaptability [1]. Investigating the growth and development of sheep to improve the productivity and efficiency of mutton production is essential for bolstering the competitiveness of the meat sheep industry [2,3].

Nowadays, classical breeding methods are supported by various molecular genetics tools to accelerate the achievement of breeding goals [4]. An InDel locus can produce a polymorphism marker with a certain length, and it is one of the most abundant variation types in animal and plant genomes [5]. It has been demonstrated in numerous studies that Indel polymorphisms are associated with growth traits in domestic animals [6,7,8,9]. Marker-assisted selection (MAS) is an alternative to DNA mutations, which is independent of the microenvironment [10]. InDel genetic markers are numerous, accurate and highly stable, and they also have STR and SNP characteristics. Among the main genetic variants, InDel detection is the most convenient, as it does not require special equipment or technical conditions. Molecular marker technology is continuously evolving, and is addressing the shortcomings of traditional breeding methods, such as a long cycle and the unstable inheritance of good traits. Consequently, breeding improvement can be advanced more effectively. Additionally, InDel detection is widely used in the screening of key economic traits in animals [11]. Based on the feasibility of this detection, we could use the MAS method to continue to improve the growth traits of the Chinese sheep population.

Sheep have long been an important part of the global agricultural economy [12]. There are abundant local sheep breeds in China. Tong sheep (TS), Hu sheep (HS), Small-Tail Han sheep (STHS) and Lanzhou fat-tailed sheep (LFTS) are all representative breeds of high-quality sheep. Some previous studies indicated that Mongolian sheep were the origin of these four sheep [13,14]. Tong sheep, a breed of fat-tailed sheep in China, are distinguished by their high-quality mutton, semi fine wool, and pearl-like suede. The fat-tailed characteristic makes it highly adaptable to harsh environments [15]. Lanzhou fat-tailed sheep, an excellent sheep breed with good meat productivity, crude feed tolerance, and high disease resistance, are mainly distributed in Gansu province [16]. Hu sheep have a number of excellent characteristics, such as good meat quality, hyper-prolificacy, and beautiful wavy lambskins. Nowadays, the breed has developed breed-specific features and is distributed across almost all of China [17]. Small-tailed Han sheep, the typical mutton breed in northern China, are known for their high lambing rate, good quality of fur and meat, and strong resistance to diseases [18]. It is worth noting that sheep can not only produce economic products such as mutton, wool, milk and sheep skin, but can also be used as a large circadian animal model for medical research [19].

In mammals, the circadian clock is composed of the central clock in the suprachiasmatic nucleus (SCN) of the brain and peripheral clocks found throughout nearly every tissue and organ system. The core clock genes primarily consist of clock gene encoding positive regulators (circadian locomotor output cycles kaput, *CLOCK*), hydrocarbon receptor nuclear translocation protein-like 1 gene (brain and muscle Arnt-like 1, *BMAL1*), cycle gene encoding negative regulators (period, *PER*) and the cryptochrome gene (cryptochrome, *CRY*) [20]. The *CLOCK* gene includes a protein, known as CLOCK, which engages in interactions with various other proteins, notably BMAL1, for the purpose of governing the circadian rhythm. In concert, these proteins adhere to distinct sections of DNA, thereby aiding in the regulation of the functionality of other genes. This orchestration instigates oscillations in protein levels, ultimately contributing to forming a stable cycle within the organism [21,22,23]. The synchronized expression of circadian genes is essential for the organization of the 24-hr cycle [24]. Chen et al. found significant age-dependent rhythmic expression of the *PER1* and *PER2* gene in the prehuman prefrontal cortex [25].

Circadian clocks are typically expressed in proliferating tissues, including the oral cavity, gastrointestinal mucosa, skin, and bone marrow [26]. Extensive research has been conducted on the relationship between ovine circadian genes and reproductive performance, indicating that these genes regulate hormone secretion, metabolism, growth, and reproduction in sheep [27]. *CLOCK* and *PER3* genes are core clock genes that play pivotal roles in skeletal and muscle development, as well as other growth and developmental processes [28,29,30,31,32]. The *CRY2* gene was closely associated with reproductive performance and carcass traits in sheep. Variations in the *CRY2* gene was significantly associated with the number of lambs born in the first and third litters of Australian white sheep [33]. Recent studies have suggested that the *CRY2* gene variant was significantly related to 12 carcass traits, including gross weight, ribeye, high rib, thick flank [34]. Polymorphisms in the *CRY1* InDel locus were significantly associated with twenty carcass traits, such as slaughter weight, limb weight, and belly meat weight [35]. Furthermore, Zhou et al. found that the circadian clock *RORα* in skeletal muscle regulates growth and development, and was significantly correlated with body height, hip cross height, and body length [36].

Our study aims to identify potential InDels of the *CLOCK*, *PER2*, *PER3*, *CRY1*, and *CRY2* genes in four Chinese sheep breeds, and to further analyze their correlation with sheep growth traits. The purpose is to provide a relevant theoretical basis for breeding sheep breeds with excellent growth performance.

## 2. Materials and Methods

### 2.1. Ethics Statement

The animal protocols and experimental design adhere to local animal welfare legislation and institutional guidelines. Furthermore, the use of animal testing received approval from the Institutional Animal Care and Use Committee at Northwest A & F University (IACUC-NWAFU) in China.

### 2.2. Animal Samples, Data Collection, DNA Extraction and Genomic DNA Pools Construction

A comprehensive evaluation was conducted involving a total of 578 samples derived from four local sheep breeds. The breeds examined include Tong sheep (TS), Hu sheep (HS), Small-Tail Han sheep (STHS), and Lanzhou fat-tail sheep (LFTS). Blood samples and body measurements were obtained from four local sheep breeds. All animals utilized were adults, in good health, and unrelated. All animals within a given breed were managed in the same way, and an adequate supply of feed was ensured based on the total metabolic rate (TMR). The growth traits of the sheep were measured, utilizing the measuring tape, including body weight, body height, body oblique length, rump width, sacrum height, back height, hip height, chest depth, chest girth, cannon girth, cross height and so on. Additionally, blood samples were aseptically obtained from the jugular vein of all individuals using EDTA vacutainers. Subsequently, genomic DNA was extracted from the collected blood samples, and the quality and purity of the extracted DNA were assessed using a Nano Drop 10,000 (Thermo Scientific, Waltham, MA, USA). All DNA samples were diluted to 50 ng/µL and subsequently stored at a temperature of −20 °C, as outlined by Gao et al. [37]. The DNA mixture was generated through the random selection of 20 individuals from each breed, ensuring that the DNA was combined in equal proportions.

### 2.3. Primer Design, PCR Amplification, and InDel Genotyping

The primer design for gene amplification was based on the National Center for Biotechnology Information (NCBI) database’s sheep genome sequences: NC_056059.1 (*CLOCK*, chromosome 6), NC_056065.1 (*PER3*, chromosome 12), NC_056054.1 (*PER2,* chromosome 1), NC_056056.1 (*CRY1,* chromosome 3) and NC_056068.1 (*CRY2,* chromosome 15), of the sheep genome (*Ovis aries*). All primers for InDels of circadian clock genes in essential positions were designed by the NCBI primer-blast (Primer designing tool (nih.gov)). They were produced by Biotech Bioengineering Co. (Shanghai, China). For the PCR, we used 15 μL of the reaction mixture, containing 7.5 μL of 2× San Tap PCR Mix, 1.0 μL of each primer (forward and reverse primer), 0.5 μL of DNA template, and 6 μL of ddO. A touch-down PCR cycling was run for each mixture: initial denaturation at 95 °C for 5 min; followed by 15 cycles of 30 s at 95 °C, 30 s at 60 °C, and 30 s at 72 °C; followed by another 25 cycles of 30 s at 95 °C, 30 s at 53 °C, and 30 s at 72 °C, with a final extension step of 10 min at 72 °C. Subsequently, the PCR products were identified by electrophoresis on a 3.0% agarose gel at 150 V, 200 mA, and 1–1.5 h. The sequencing of PCR products was performed by Biotech Bioengineering Co. (Shanghai, China).

### 2.4. Statistical Analysis of Population Genetics

Microsoft Excel software was used to collate all individual genotypes at each site and calculate genotype frequency and allele frequency. The Sanger Atlas sequence data and the reference genome were compared with Bioedi [38]. The Hardy–Weinberg equilibrium (HWE), homozygosity (Ho), heterozygosity (He), effective allele numbers (Ne), and polymorphism information content (PIC) were calculated using the online website http://www.Msrcall.com (accessed on 26 July 2022) [39,40]. Chi-squared tests of different genotypic frequencies and breeds were performed by “χ^2^ calculator”. According to the statistical results, one-way analysis of variance (ANOVA) was performed to analyze the association between body measurements and different genotypes of four sheep breeds using SPSS 25.0 software. The above results were displayed as “mean ± standard error” (Mean ± SE). *p* < 0.05 indicated that the difference was significant, and *p* < 0.01 indicated that the difference was extremely significant. The Bonferroni test was performed for multiple comparisons.

## 3. Results

### 3.1. Indel Genotyping and Sequencing

**Figure 1:**
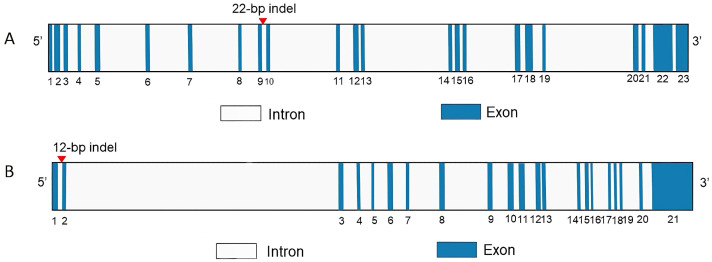
The position and gene structure map of *PER3* (**A**) and *CLOCK* (**B**).

**Figure 2:**
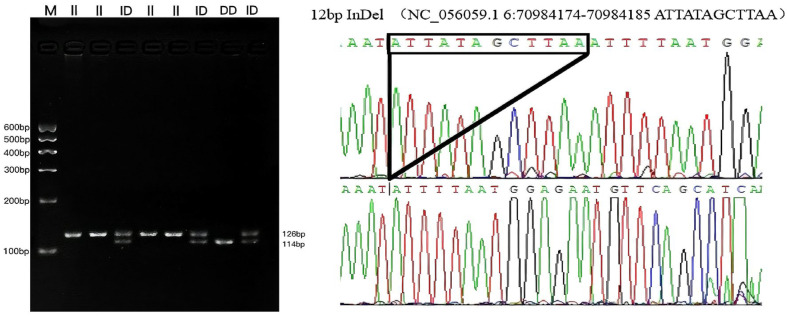
The electrophoresis pattern and sequence chromatograms of the CLOCK-P13.

**Figure 3:**
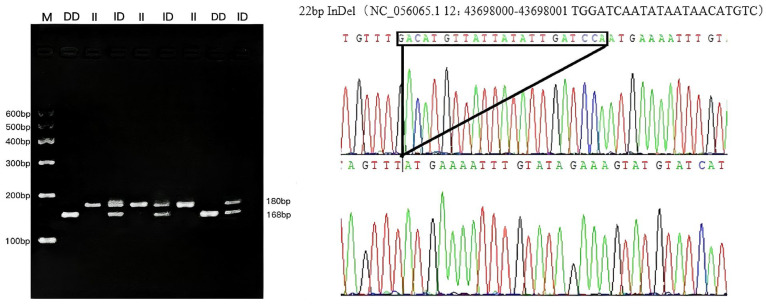
The electrophoresis pattern and sequence chromatograms of the PER3-P4.

Two InDel loci were confirmed from 23 potential loci within five genes (*CLOCK*, *PER2*, *PER3*, *CRY1* and *CRY2)* using 3% gel electrophoresis and Sanger sequencing (Table S1). One InDel locus was detected in *CLOCK* on chromosome 6 (P13-Del-12-bp) and one InDel locus was identified in *PER3* on chromosome 12 (P4-Ins-22-bp) (Table 1). Sanger sequencing revealed that 12 bases were missing in *CLOCK*, which is located at NC_056059.1 g. 70984174-70984185, with the missing bases ATTATAGCTTAA. At the *PER3* locus, 22 bases were inserted at NC_056065.1 g. 43698000-43698021, and the inserted bases were GACATGTTATTATATTGATCCA (Figure 1, Figure 2 and Figure 3). Both InDel loci are located in the intronic regions of the genes. The other 21 InDel loci showed no polymorphism in HS, TS, STHS, and LFTS.

### 3.2. Genetic Polymorphism Analysis of CLOCK Gene in Hu Sheep, Tong Sheep, Small-Tail Han Sheep, and Lanzhou Fat-Tailed Sheep

The genotypic and allelic frequencies, as well as other genetic parameters, associated with the *CLOCK* gene InDel locus, were calculated to determine the genotype distribution in HS, TS, STHS, and LFTS (Table 2). However, only HS had the *CLOCK* 12-bp deletion mutation.

As can be seen from the data, in these four breeds, the “I” allele was more frequent than the “D” allele. Additionally, the PIC in HS, TS, STHS, and LFTS were 0.352, 0.351, 0.273, and 0.331, respectively, which demonstrated a moderate degree of polymorphism (0.25 < PIC < 0.5), indicating that the polymorphism of the *CLOCK* gene is substantial. The P13-Del-12-bp locus was in HWE (*p* > 0.05).

### 3.3. Genetic Polymorphism Analysis of PER3 Gene in Hu Sheep, Tong Sheep, Small-Tail Han Sheep, and Lanzhou Fat-Tailed Sheep

The genotype and allele frequencies and other genetic parameters associated with the *PER3* gene InDel locus were calculated (Table 3). TS, STHS, and LFTS had the *PER3* 22-bp insertion mutation. In these four breeds, the frequency of the II genotype was observed to be higher compared to the other genotypes. In addition, the allele frequency of “I” was more frequent than that of “D”. The frequency of the II genotype in TS was observed to be greater than that in the three other evaluated breeds. Moreover, the 22 bp InDel genotype frequency was found to be in accordance with HWE (*p* > 0.05) in HS, TS, STHS, and LTHS. Based on PIC values, genetic diversity was low in TS (PIC = 0.219), whereas moderate in HS, STHS, and LFTS (0.25 < PIC < 0.5).

### 3.4. Association Analysis of Growth Traits in Hu Sheep with the CLOCK Gene

**Figure 4:**
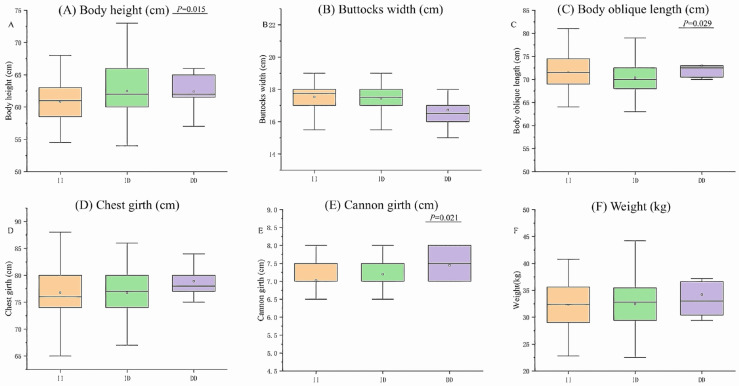
Associations of the 12 bp loci within the *CLOCK* gene with growth traits in Hu Sheep. (**A**) Body height (cm), (**B**) Buttocks width (cm), (**C**) Body oblique length (cm), (**D**) Chest girth (cm), (**E**) Cannon girth (cm), (**F**) Weight (kg).

The results of the sequencing showed that only HS had the *CLOCK* 12-bp deletion mutation. Therefore, an association study was performed to assess the effect of polymorphism on growth traits in HS. In Hu sheep, the 12-bp InDel of *CLOCK* was significantly associated with body height, body oblique length and cannon girth traits (*p* = 0.015, *p* = 0.029, and *p* = 0.021; Figure 4). The body oblique length and cannon girth traits of DD genotypes were greater than in the ID and II genotypes.

### 3.5. Association Analysis of Growth Traits in Tong Sheep and Small-Tail Han Sheep with the PER3 Gene

**Figure 5:**
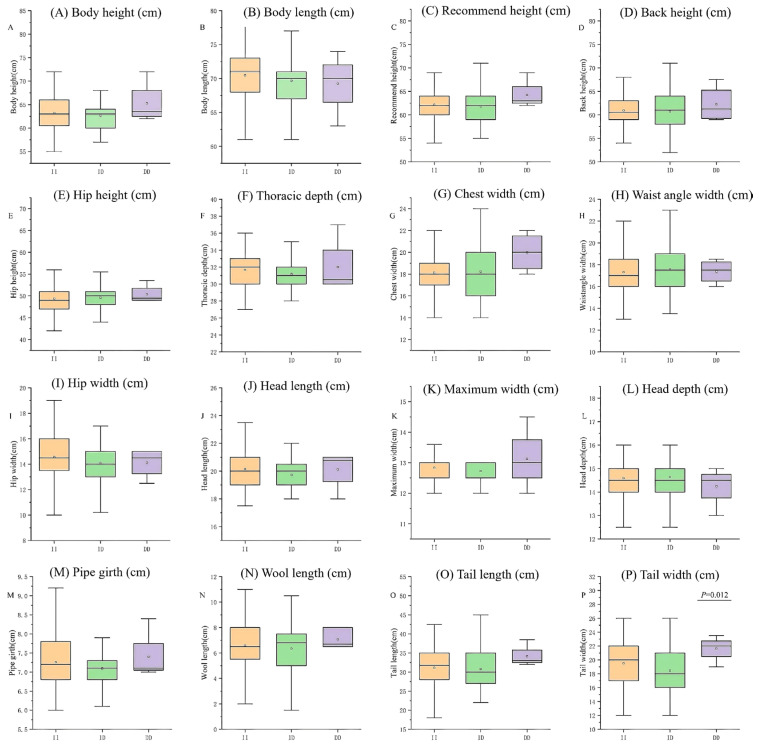
Associations of the 22 bp loci within the *PER3* gene with growth traits in Tong sheep. (**A**) Body height (cm), (**B**) Body length (cm), (**C**) Recommend height (cm), (**D**) Back height (cm), (**E**) Hip height (cm), (**F**) Thoracic depth (cm), (**G**) Chest width (cm), (**H**) Waist angle width (cm), (**I**) Hip width (cm), (**J**) Head length (cm), (**K**) Maximum width (cm), (**L**) Head depth (cm), (**M**) Pipe girth (cm), (**N**) Wool length (cm), (**O**) Tail length (cm), (**P**) Tail width (cm).

**Figure 6:**
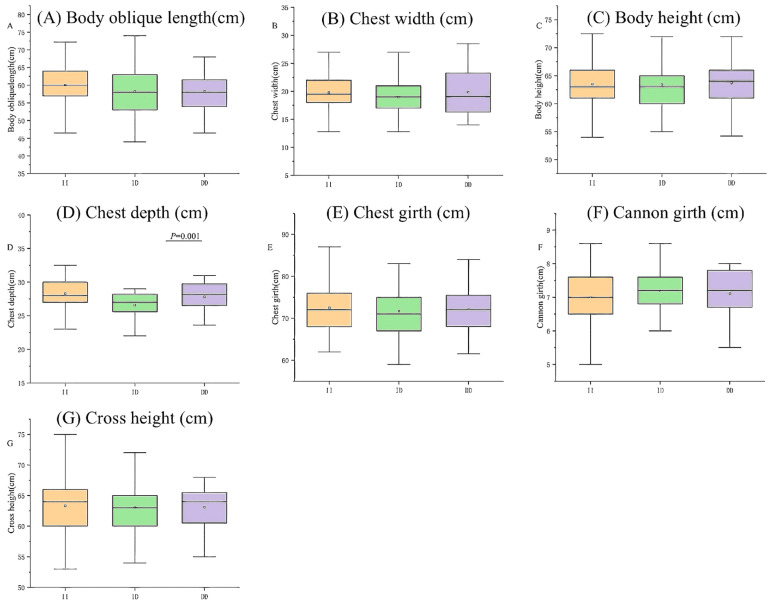
Associations of the 22 bp loci within the *PER3* gene with growth traits in Small Tail Han Sheep. (**A**) Body oblique length (cm), (**B**) Chest width (cm), (**C**) Body height (cm), (**D**) Chest depth (cm), (**E**) Chest girth (cm), (**F**) Cannon girth (cm), (**G**) Cross height (cm).

The results of sequencing showed that the 22-bp insertion loci of *PER3* were detected in TS and STHS. Subsequently, the association between the *PER3* InDel locus and the growth traits of TS and STHS were examined. We conducted a series of experiments, and we propose that the 22-bp InDel of *PER3* was significantly associated with tail width in TS (*p* = 0.012, Figure 5) and chest depth in STHS (*p* = 0.001, Figure 6). TS, with the DD genotype, had greater tail width than those with the ID and II genotypes. Notably, in STHS, the II genotype had greater chest depth than those with the ID and DD genotypes.

## 4. Discussion

Our study shows that InDels of the core clock genes *CLOCK* and *PER3* were identified for the first time in four Chinese sheep breeds using sequencing technology. Due to the precision and stability of InDel markers, the *CLOCK* and *PER3* genes can be considered as key genes for affecting growth traits in sheep. This result provides a valuable reference for genetic breeding improvement in sheep.

Existing research indicates that the clock gene *RORα* in skeletal muscle regulates growth and development, and that this gene is significantly associated with body height, hip cross height, and body length. The clock gene *RORα*, which encodes ROR, is present in skeletal muscle. According to a study, the P11-28-bp deletion fragment of the *RORα* gene in goats was significantly associated with body length, hip cross height, and body height [41]. Bone remodeling is a homeostatic function [24], suggesting that the circadian clock could control bone mass. Mice lacking PER and CRY, or lacking PER genes in osteoblasts, display high bone mass [42]. The 9 bp deletion fragment of *PER1* gene in sheep was associated with body height, cross height, chest depth, body length index, and cannon girth [43]. In mammals, it has been proved that the variation of circadian clock genes affect growth in mice. Current research on circadian clock genes in sheep focus primarily on reproductive performance and lamb skin quality, while correlation analysis of growth traits has received less attention.

To demonstrate the relationship between clock genes and growth traits, we conducted genotyping for *PER2, PER3, CRY1, CRY2* and *CLOCK* in four local sheep breeds. However, we only identified polymorphic loci in the *PER3* and *CLOCK* genes. The results indicated that there was a 12-bp deletion (P13-Del-12-bp) of *CLOCK* in HS and a 22-bp insertion (P4-Ins-22-bp) of *PER3* in TS, STHS and LTHS. The polymorphic loci of the core circadian clock genes were less typed on 578 sheep in this experiment. We analyzed that this is because most of the core components of the molecular clock maintain rhythmicity in the SCN and peripheral tissues [44]. In some instances, the circadian system genes are evolutionarily conserved [45]. Many biological processes exhibit regular fluctuations throughout the 24-h day, while almost all tissues and organs coordinate such fluctuations and shows a stable state [46]. For instance, in the absence of environmental cues (such as light), the clock can continue to operate with exceptional precision, stability, and persistence [36].

The P13-Del-12-bp locus of *CLOCK* was detected in four breeds, but after ANOVA analysis of the growth traits of these four breeds, it was found that it was only significantly associated with body height, body oblique length and cannon girth traits in HS (*p* < 0.05). The body height and cannon girth of individuals with DD and ID genotypes were significantly greater than those with the II genotype, and the body oblique length of the DD genotype was larger than that of the ID and II genotype, which suggested that the deletion of this segment could improve the growth and development rate of HS. However, the number of DD genotype individuals is the smallest, which suggests that the selection of HS is a preference for thin-bone type individuals. HS are mostly breeding ewes, so the selection of reproductive performance may ignore the selection of growth traits. There was no significant association between the P13-Del-12-bp locus and the growth traits of TS and STHS. It is evident that the InDel locus within the same gene varies across different sheep breeds, indicating that the gene is breed specific.

The P4-Ins-22-bp locus of *PER3* in TS and the tail width of II and DD genotypes were significantly higher than that of the ID genotype (*p* < 0.05), while the chest depth of the II and DD genotypes in STHS were significantly higher than that of the type ID genotype (*p* < 0.01), with no significant association with growth traits in Hu sheep (*p >* 0.05). By analyzing the effect of tail width on TS, the tail width with the DD genotype was greater than that with the ID and II genotypes. It can be inferred that the insertion of this segment may cause the growth of TS to be slow. When analyzed together with the data from the STHS, it was found that the chest depth of the II genotype was greater than that of the ID and DD genotypes, indicating that the insertion of this segment promotes the growth and development of STHS. The P4-Ins-22-bp locus has opposite effects on tail width in TS and STHS, once again confirming the species specificity of the gene.

In this study, these two loci were in Hardy–Weinberg equilibrium (HWE), demonstrating that our sample of four local sheep breeds was sufficiently robust. This indicates that the majority of loci in the sheep of these four breeds remain in dynamic equilibrium under the influence of artificial selection, migration, and genetic drift, exhibiting moderate polymorphism and high adaptability in the face of environmental changes and thereby ensuring that the study samples are representative of the populations. The dependability of the study sample enables the next step of association with growth traits in sheep [15]. On the other hand, the expected heterozygosity (He) describes the expected number of heterozygous genotypes under Hardy–Weinberg equilibrium and serves as an indicator of genetic variation in a population. Additionally, the number of effective alleles (Ne) and the polymorphic information content (PIC) can also be used to assess the genetic diversity of a population [11]. The P13-Del-12-bp InDel locus in HS, STHS, and LTHS, and the P4-Ins-22-bp InDel locus in four sheep showed moderate genetic polymorphisms (0.25 < PIC < 0.5). The greater the value, the richer the genetic diversity of the population, and the corresponding potential for selection will be greater. Our data showed that the P1-Del-12-bp locus of the *CLOCK* gene had high genetic diversity in HS, which could be attributed to the introduction of HS from south to north, as well as its high genetic diversity due to a diverse geographic environment and long-term breeding adaptation [47]. Apart from this, the PIC value indicated that the P4-Ins-22-bp locus on TS was 0.219, and exhibited low genetic polymorphisms (PIC < 0.25). We infer that the TS of this test may not have been subjected to extensive breeding selection. (Table 2 and Table 3). Regarding the limitation of this, it could be argued that the long-term isolation of the TS with more inbreeding and limited genetic interactions resulted in a unique genetic background and lower genetic diversity, resulting in a relatively homogeneous pedigree. Based on this, it is suggested that we should increase the selection intensity of the *PER3* gene associated with growth traits of TS and increase the population of TS.

The five InDel loci associated with growth traits in HS, TS, and STH, as identified in this experiment, were all located in the intronic region of the genes. Introns enable genes to produce a variety of different splicing modes. In eukaryotes, there are many cis-acting elements in introns. These elements are involved in transcriptional regulation and play a role as promoters, enhancers or suppressors. In recent years, there have been several demonstrations of the ability of introns to enhance gene expression in a variety of organisms, including animals, plants and microorganisms [48]. Concurrently, introns can not only accelerate the rate of transcription and translation, but also exert a repressive influence on the expression of some genes [49]. This can explain the disparate effects of the identical InDel loci on the growth traits of the four breeds of sheep in this experiment. It is also postulated that the insertion or deletion of the InDel loci altered the sequence of the introns, which in turn affected the rate of transcription and translation of the genes, and thus played a role in affecting the growth and development of sheep. A limitation of this study is that there were only 57 LTHS, as this breed is mainly distributed in Gansu province. Due to the limited amount of breeding and the plan of conservation programs for LTHS, the sample size we collected was relatively small, which may mean that the sequencing results for LTHS are not very representative [13]. However, it is undeniable that this study provides direction and a reference basis for further in-depth exploration of the molecular mechanism of clock genes affecting sheep growth traits.

## 5. Conclusions

In conclusion, our study demonstrated that the 12 bp InDel of the *CLOCK* gene was significantly associated with body height, body oblique length and cannon girth in Hu sheep. The 22 bp InDel of the *PER3* gene was significantly associated with tail width in Tong sheep and chest depth in Small-Tail Han sheep. These findings suggest that the *CLOCK* and *PER3* genes could be used as candidate genes for genetic breeding of sheep growth, providing a theoretical foundation and support for sheep selection and conservation purposes.

## Figures and Tables

**Table 1:** PCR primer sequences of the sheep CLOCK genes.

Genes	Variant ID	Primer Names	Primer Sequences (5′–3′)	Product Sizes (bp)	Notes
*PER3*	rs600537720	P4-Ins-22-bp	F: CAATTTCCCATGATACATAC R: TCAGCTTTACATTAGTCCTT	168	Polymorphism
*CLOCK*	rs604230640	P13-Del-12-bp	F: AGTTCTGTGGGTGAAAGTAT R: AGGCTGAACATTCTCCATTA	126	Polymorphism

**Table 2:** Genetic parameters of the 22 bp loci within *PER3* gene in four sheep breeds.

Breeds	Loci	Sizes	Genotypic Frequencies			Allelic Frequencies		Population Parameters			HWE	
		N	II	ID	DD	I	D	Ho	He	Ne	PIC	*p*-Value
HS	P13-Del-12-bp	192	0.344	0.609	0.047	0.648	0.352	0.544	0.456	1.839	0.352	0.649
TS	P13-Del-12-bp	156	0.417	0.468	0.115	0.651	0.349	0.546	0.454	1.833	0.351	0.647
STHS	P13-Del-12-bp	173	0.665	0.260	0.075	0.795	0.205	0.674	0.326	1.484	0.273	0.507
LTHS	P13-Del-12-bp	57	0.456	0.491	0.052	0.702	0.298	0.582	0.418	1.719	0.331	0.609

**Table 3:** Genetic parameters of the 22 bp loci within *PER3* gene in four sheep breeds.

Breeds	Loci	Sizes	Genotypic Frequencies			Allelic Frequencies		Population Parameters			HWE	
		N	II	ID	DD	I	D	Ho	He	Ne	PIC	*p*-Value
HS	P4-Ins-22-bp	192	0.510	0.385	0.104	0.703	0.297	0.583	0.418	1.717	0.330	0.608
TS	P4-Ins-22-bp	156	0.731	0.244	0.026	0.853	0.147	0.749	0.250	1.335	0.219	0.417
STHS	P4-Ins-22-bp	173	0.557	0.305	0.138	0.699	0.301	0.579	0.421	1.727	0.332	0.612
LTHS	P4-Ins-22-bp	57	0.579	0.351	0.070	0.754	0.246	0.629	0.371	1.590	0.302	0.558

## Data Availability

Data are contained within this manuscript and Supplementary Materials.

## Supplementary Materials

The following supporting information can be downloaded at: https://www.mdpi.com/article/10.3390/vetsci12010039/s1, Table S1: PCR primer sequences of the sheep CLOCK genes.
