# Supplementary material for: Detection of Insertion/Deletions (InDel) Within Five Clock Genes and Their Associations with Growth Traits in Four Chinese Sheep Breeds
Source: Vet Sci. 2025 Jan 9;12(1):39. doi: 10.3390/vetsci12010039 (PMC11769414; doi:10.3390/vetsci12010039)
Supplement: Supplementary file 1 [file vetsci-12-00039-s001.zip › vetsci-3369254-supplementary.pdf]

Supplementary Table S1: PCR primer sequences of the sheep CLOCK genes.

Genes	Variant ID	Primer names	Primer sequences(5'-3')	Product sizes (bp)	Notes
PER2	rs1087793953	P1-Ins-11-bp	F: CTACACGCCAAGCCCCACCA<br>R: GGCCCGTGGTCTGAGCAAGT	137	No polymorphism
PER2	rs1090114677	P2-Ins-8-bp	F: GCCACCGCCTTTGTTCCCTG<br>R: TGCCTGAGCGCAGACTTCCT	157	No polymorphism
PER2	rs605418044	P3-Del-6-bp	F: GCGTCGACTCTTCGGAGAAA<br>R: GCAGGCATCTGGTAGGTCATC	151	No polymorphism
PER3	rs1088672257	P5-Del-6-bp	F: ACGTTGGCTATCTCCTCCCT<br>R: AAGAACCCTGTGCCTTGTTT	163	No polymorphism
PER3	rs1092500098	P6-Del-6-bp	F: TAATACCTAGCTCCTGTTCT<br>R: GATATGGGTGGCTAATACTT	156	No polymorphism
PER3	rs1087082193	P7-Del-18-bp	F: TCCCGTCGTTTCCATCTTTG<br>R: TGATCTTCCCAGGTCGGCTA	106	No polymorphism
CRY1	rs604061994	P8-Del-15-bp	F: GTACACTACCATCAACAGCAAC<br>R: ATCCAAGTCTAACTTGTCCTAA	151	No polymorphism
CRY1	rs592042250	P9-Del-8-bp	F: ATGCATGTTTATGATAAATCTC<br>R: ACTGAATGAGGATAGCCATA	160	No polymorphism
CRY2	rs597171453	P10-Del-6-bp	F: AGTGACTCCCATTGTTGACG<br>R: CCACTGACCTGCTCTTCTGC	116	No polymorphism
CRY2	rs1087839609	P11-Ins-6-bp	F: TACTGCTGTTACTATTACCATCG<br>R: GACCCAGTGAGAACCCTTTC	107	No polymorphism
CRY2	rs1085959825	P12-Del-6-bp	F: CAGTCCCCAAGCAAGAAGAG<br>R: CAGTGACCGCCTGGAAAGAT	134	No polymorphism
CLOCK	rs596223672	P14-Del-10-bp	F: AATCATCCTAACCCCTAATC<br>R: TAGCGTGTATAATAATCCTCAA	118	No polymorphism
CLOCK	rs1085437460	P15-Ins-6-bp	F: TGCAAGCATGAGTAGGATCT<br>R: TCTTACTCTGAGCAGCCACA	161	No polymorphism
CLOCK	rs602045685	P16-Del-8-bp	F: TTGAGGATTATTATACACGCTA<br>R: ATGCAACTAGGACTTTGGTA	149	No polymorphism
CLOCK	rs596822075	P17-Del-7-bp	F: GTCTTCCCTGTATCCTTTTG<br>R: CATTTGTAATCAGTCAAGCT	135	No polymorphism
CLOCK	rs605411101	P18-Del-9-bp	F: CTACCGCTACAATTCTATAA<br>R: ATTATTTCACATACACTGGC	117	No polymorphism
CLOCK	rs587983767	P19-Del-6-bp	F: AGTTTGAGCATCCTGACTAG<br>R: CTAGATCACAGGTAACTCCC	111	No polymorphism
CLOCK	rs590512949	P20-Del-8-bp	F: GGGGAACAATACTTAACAAA	152	No polymorphism

			R: ACGTAGATAATACCCCAAAC		
CLOCK	rs603672163	P21-Del-8-bp	F: AAATCTCAGTACCCAGCAGT<br>R: AGAGCAGATGGGATTCAGAG	139	No polymorphism
CLOCK	rs1092231495	P22-Del-19-bp	F: AAGTAAGACTAAATAAGCAGC<br>R: CCAAGCCAAGGATAGAACTG	144	No polymorphism
CLOCK	rs604230640	P23-Del-12-bp	F: TAATGTCAGTTCTGTGGGTG<br>R: AGTAGGTATCTATGAAGCAG	151	No polymorphism
